# Type I Interferon Receptor on NK Cells Negatively Regulates Interferon-γ Production

**DOI:** 10.3389/fimmu.2019.01261

**Published:** 2019-06-04

**Authors:** Amanda J. Lee, Firoz Mian, Sophie M. Poznanski, Michele Stackaruk, Tiffany Chan, Marianne V. Chew, Ali A. Ashkar

**Affiliations:** Department of Pathology and Molecular Medicine, McMaster Immunology Research Centre, McMaster University, Hamilton, ON, Canada

**Keywords:** NK cells, type I IFN, IFN-γ, HSV, Human NK cells

## Abstract

NK cells are a key antiviral component of the innate immune response to HSV-2, particularly through their production of IFN-γ. It is still commonly thought that type I IFN activates NK cell function; however, rather than requiring the type I IFN receptor themselves, we have previously found that type I IFN activates NK cells through an indirect mechanism involving inflammatory monocytes and IL-18. Here, we further show that direct action of type I IFN on NK cells, rather than inducing IFN-γ, negatively regulates its production during HSV-2 infection and cytokine stimulation. During infection, IFN-γ is rapidly induced from NK cells at day 2 post-infection and then immediately downregulated at day 3 post-infection. We found that this downregulation of IFN-γ release was not due to a loss of NK cells at day 3 post-infection, but negatively regulated through IFN signaling on NK cells. Absence of IFNAR on NK cells led to a significantly increased level of IFN-γ compared to WT NK cells after HSV-2 infection *in vitro*. Further, priming of NK cells with type I IFN was able to suppress cytokine-induced IFN-γ production from both human and mouse NK cells. We found that this immunosuppression was not mediated by IL-10. Rather, we found that type I IFN induced a significant increase in Axl expression on human NK cells. Overall, our data suggests that type I IFN negatively regulates NK cell IFN-γ production through a direct mechanism *in vitro* and during HSV-2 infection.

## Introduction

Natural killer (NK) cells are innate lymphocytes that, when appropriately activated, are capable of killing virally infected and malignant cells (1). NK cells are an important component of the innate antiviral response as they can directly eliminate infected cells and release pro-inflammatory cytokines that can alter the microenvironment and activate other immune cells (1). During virus infection, NK cell release of IFN-γ as part of the innate response can induce nitric oxide from macrophages to inhibit virus replication as well as promote maturation of dendritic cells (DCs) and polarize Th1 cell adaptive immune responses (2–4). IFN-γ is a critical cytokine as part of the immune response against Herpes Simplex Virus type 2 (HSV-2) infection. *Ifny*^−/−^ mice have significantly decreased survival after HSV-2 infection, a lifelong virus infection that causes genital ulcer disease and increases the risk of HIV acquisition (5–8).

NK cell function can be mediated through the ligation and signaling of activation and inhibitory receptors, wherein an overwhelming activation signal will enable their effector functions (9). As NK cells mature, their progress can be determined by examining their expression of CD27 and CD11b (10, 11). Immature NK cells start out as a double negative population lacking expression of both CD27 and CD11b (CD27–CD11b–) (11). As they mature, they gain expression of CD27 (CD27+CD11b–) and progress to a double positive population with expression of both CD27 and CD11b (CD27+CD11b+) (11). The most mature subset of NK cells has lost expression of CD27, but maintains expression of CD11b (CD27–CD11b+) (11). Groups have suggested that NK cells expressing CD27 are highly cytotoxic and cytokine producers, conversely, others have suggested that CD11b+ NK cells have the highest effector potential (10, 11).

Cytokines within the local environment can also regulate NK cell antiviral function, where cytokines such as type I interferon (IFN), IL-12, IL-15, and IL-18 are known to activate NK cell function, particularly IFN-γ production (9). Type I IFN has been shown to be critical for NK cell activation during virus infection, though the mechanism by which type I IFN activates NK cells is still under debate. We have previously shown that type I IFN does not activate NK cells directly during HSV-2 infection (12). Instead, type I IFN induces inflammatory monocytes to release IL-18, which then activates IFN-γ production from NK cells (12). Further, we also found that type I IFN receptor on NK cells was required to negatively regulate their IFN-γ release, as transfer of *Ifnar*^−/−^ NK cells into alymphoid mice led to significantly increased and sustained levels of IFN-γ production in comparison to WT NK cells during HSV-2 infection (12). This suggested to us that type I IFN signaling on NK cells, rather than activating them, negatively regulates their ability to produce IFN-γ. Thus, type I IFN can potentially differentially modulate NK cell function through direct or indirect mechanisms.

Type I IFN consists of a family of structurally similar cytokines, including a single IFN-β and 13–14 subtypes of IFN-α, that all signal through the same type I IFN receptor (IFNAR) (13). Type I IFN, though considered to be a potent antiviral cytokine, has also been found to negatively regulate immune cells. In particular, Teijaro et al. found that blocking the type I IFN receptor during chronic LCMV infection was able to restore IFN-γ production, suggesting that type I IFN can suppress antiviral effector functions (14). Others have found that type I IFN can negatively regulate NK cell function, where exogenous type I IFN was able to suppress NK cell IFN-γ release (15). Further, a late wave of IFN-β during a *Listeria monocytogenes* infection suppressed NK cell function (16). In an intracellular bacterial infection, type I IFN led to IL-10 production, which contributed to suppression of an IFN-γ response (17). Type I IFN has also been shown to upregulate TAM (Tyro 3, Axl, and Mer) receptor expression and can co-opt the type I IFN receptor signaling cascade to induce expression of suppressor of cytokine signaling (SOCS) proteins (18). In humans, administration of pegylated IFN-α therapy to hepatitis C patients leads to a significant reduction in IFN-γ positive NK cells in the peripheral blood of these patients (19, 20).

In this study, we found that rather than activating NK cells, NK cells required the type I IFN receptor to negatively regulate their IFN-γ production during HSV-2 infection and during IL-15 and IL-18 stimulation. During infection, NK cell IFN-γ levels peak at day 2 post-infection and are subsequently negatively regulated at day 3 post-infection. We found that culturing *Ifnar*^−/−^ NK cells with *Rag2*^−/−^γ*c*^−/−^ cells during HSV-2 infection led to a significant upregulation of IFN-γ in comparison to WT NK cells. Further, type I IFN pre-treatment prior to cytokine stimulation led to a significant reduction in NK cell IFN-γ production in both isolated NK cells and splenocytes, suggesting that type I IFN can both directly suppress NK cell function alone and in the presence of other immune cells. We found that type I IFN does not mediate its negative regulatory function through IL-10 induction, but did lead to a significant increase in Axl expression. Thus, type I IFN negatively regulates human and mouse NK cell IFN-γ release during HSV-2 infection and *in vitro*. In summary, our papers show a new fundamental understanding of how type I IFN directly and indirectly modulates NK cell function.

## Materials and Methods

### Mice

Six to eight week old C57BL/6 (WT) mice were purchased from Charles River Laboratory. *Ifnar*^−/−^ mice were generously provided by Dr. Laurel Lenz (University of Colorado, Boulder, CO), backcrossed onto a C57BL/6 background, and a breeding colony was established at McMaster University's Central Animal Facility (CAF). *Rag2*^−/−^γ*c*^−/−^ mice on a Balb/c background were generously provided by Dr. M. Ito (Central Institute for Experimental Animals, Kawasaki, Japan) and a breeding colony was established at McMaster University's CAF. Mice were housed in specific pathogen free conditions on a 12 h day and 12 h night cycle. All experiments were performed in accordance with Canadian Council on Animal Care guidelines and with approval from McMaster University's Animal Research Ethics Board.

### HSV-2 *in vivo* Infection

Mice were given 2 mg of Depo-Provera (medroxyprogesterone acetate) subcutaneously 5 days prior to infection. Mice were then infected with 1 × 10^4^ pfu HSV-2 333 intravaginally. Vaginal lavages were collected at baseline through to day 3 post-infection once daily. For vaginal lavage collection, 30 μL of PBS were flushed in and out of the vaginal tracts twice. Samples were centrifuged at 800 g for 5 min and supernatants were collected and assayed for protein via ELISA.

### Tissue Processing

Spleens were mechanically crushed into a single cell suspension and red blood cells were removed using an ACK lysis buffer. Vaginal tissue was isolated and processed into smaller pieces and then digested using a mixture of RPMI-1640 with 10% fetal bovine serum, 1% penicillin, 1% streptomycin, 1% L-glutamine, 1% hepes and collagenase A. Tissue was digested at 37°C for 1 h twice.

### Flow Cytometry

Mouse cells were blocked with anti-CD16/CD32 antibody to prevent non-specific binding. Extracellular surface markers were then stained with APC-conjugated anti-mouse CD45, Alexa Fluor 700-conjugated anti-mouse CD3, PE-conjugated anti-mouse NK1.1, PE-cy7-conjugated anti-mouse CD11b, Percp-conjugated anti-mouse CD27. Human cells were stained with PE-CF594-conjugated anti-human CD56, APC-H7-conjugated anti-mouse CD3, PE-conjugated anti-human Tyro3, APC-conjugated anti-human Mer, and PE-conjugated anti-human Axl. Viability was assessed using a fixable viability dye (efluor 520). Cells were fixed in 1% paraformaldehyde prior to running the samples on a BD Biosciences FACSCanto, BD LSR II, or BD LSR Fortessa. Samples were analyzed using FlowJo software.

### PBMC Isolation

Peripheral blood was collected with written-informed consent and with approval by the Hamilton Integrated Research Ethics Board at McMaster University. Peripheral blood was collected in an ACD solution A vacutainer (BD Biosciences) to prevent clotting. Blood was then diluted with 2% FBS in PBS and then separated using a Lymphoprep density gradient centrifugation method (Stem Cell Technologies). Isolated PBMCs were then used for *in vitro* assays or funneled through a CD56+ NK cell isolation kit purchased from StemCell Technologies. Cells were plated at 2 × 10^5^ cells/mL and then stimulated with 100 U of IFN-β for 12 or 18 h (as specified in the figure legends) and subsequently stimulated with either 250 ng/mL IL-15 for 24 h or examined for Axl, Mer, or Tyro3 expression via flow cytometry. Supernatants were collected from cells that were further stimulated with IL-15 and examined for IFN-γ production as discussed below.

### NK Cell Isolation

Mouse NK cells were isolated using a PE-magnetic selection kit (Stem Cell Technologies) using an PE-conjugated anti-NK1.1 antibody, with purity reaching over 89% (Supplementary Figure 1). Human NK cells were isolated using a CD56+ magnetic selection kit (Stem Cell Technologies).

### HSV-2 *in vitro* Infection

*Rag2*^−/−^γ*c*^−/−^ splenocytes were isolated and infected with HSV-2 333 at an MOI of 3 in RPMI-1640 with 1% penicillin, 1% streptomycin, 1% L-glutamine, and 1% hepes. Cells were infected for 2 h. NK cells isolated from WT or *Ifnar*^−/−^ splenocytes were co-cultured with the infected *Rag2*^−/−^γ*c*^−/−^ splenocytes at a 1:4 ratio, respectively, in RPMI-1640 with 10% fetal bovine serum, 1% penicillin, 1% streptomycin, 1% L-glutamine, and 1% hepes. Cells were cultured for 48 h. Supernatants were collected and examined for IFN-γ levels.

### Cytokine Stimulation

Splenocytes, PBMCs, or isolated NK cells (both human and mouse) were cultured in RPMI-1640 with 10% fetal bovine serum, 1% penicillin, 1% streptomycin, 1% L-glutamine, and 1% hepes. Cells were pre-treated with 100 U (or the indicated concentrations) of IFN-β for 0, 4, 6, or 12 h and then stimulated with the indicated concentrations of IL-15 or IL-18 for 24 h. Cells were also stimulated with 100 U of IFN-β for 18 or 24 h.

### ELISA

Mouse and human IFN-γ levels were measured using an R&D Systems ELISA kit. Mouse IL-10 levels were assayed using an R&D Systems ELISA kit.

### Statistical Analysis

Differences were assessed using either a student's *t*-test, a one-way ANOVA if multiple groups were being analyzed, or a two-way ANOVA if more than two groups were being analyzed with two or more independent variables. If post-statistical analysis was required, a bonferroni post-test was applied. All statistical analyses were completed using GraphPad Prism 4.0. Statistical significance is indicated as ^***^p < 0.001, ^**^p < 0.01, ^*^p < 0.05, or n.s. (not significant).

## Results

### Reduction in IFN-γ Is Not Due to a Loss in NK Cell Number at Day 3 Post-infection

**Figure 1:**
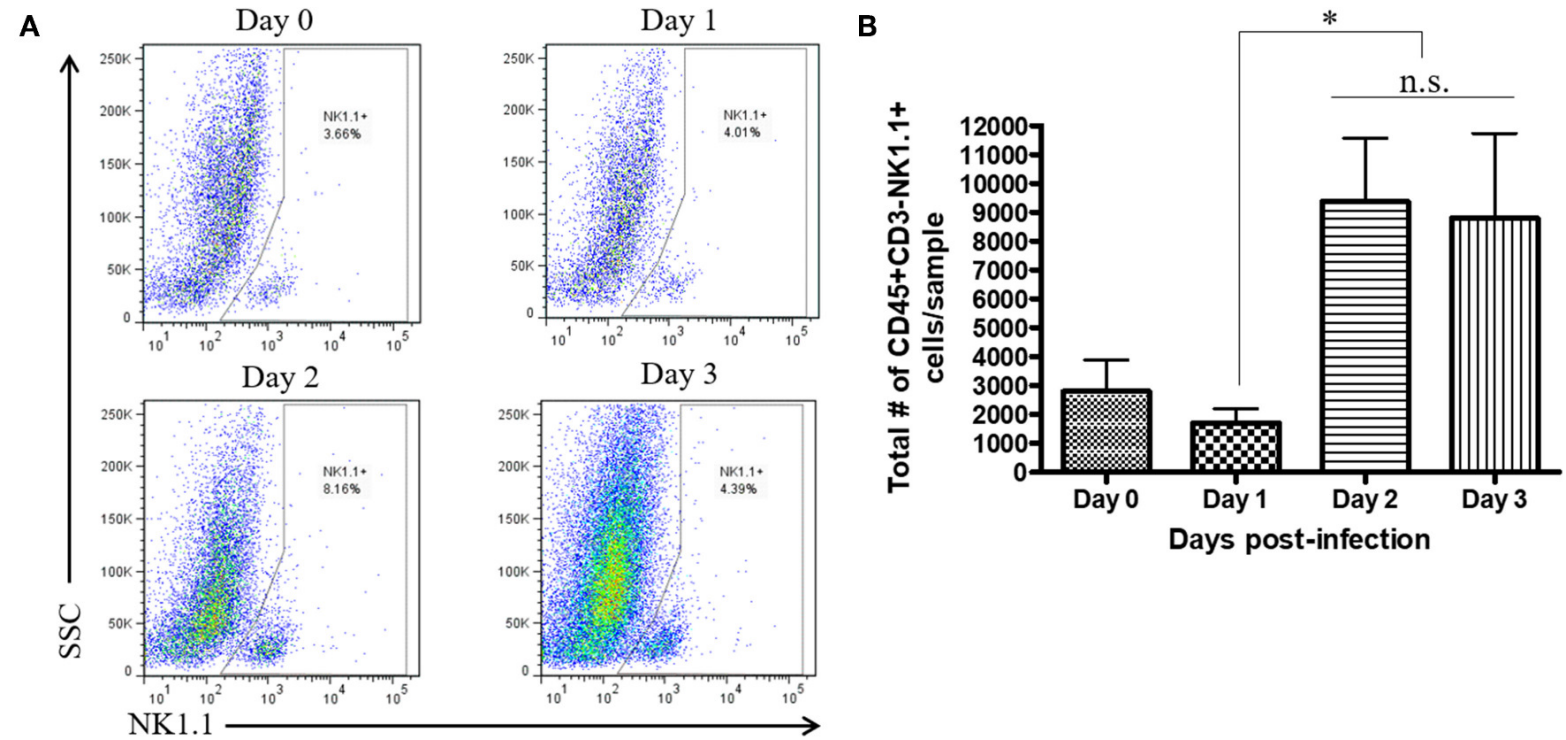
No significant difference in total number of vaginal NK cells between day 2 and day 3 post-infection. WT mice were infected with HSV-2 ivag. Vaginal tissue was isolated and processed at baseline through to d3 p.i. and stained for CD45, CD3, and NK1.1. NK cells were gated as CD45+, CD3– and NK1.1+. Representative flow plots are shown in **(A)** and total number of NK cells is shown in (**B**; *n* = 4). n.s., not significant; ^*^p < 0.05.

**Figure 2:**
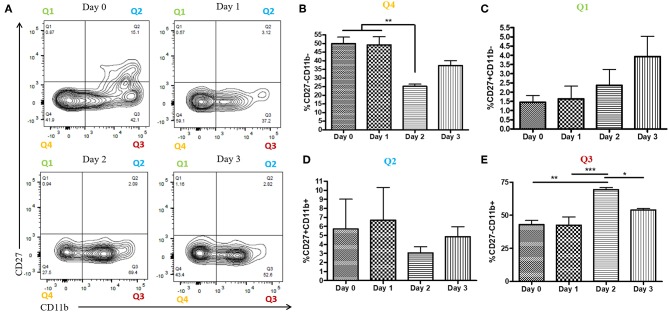
Increased proportion of CD27-CD11b+ NK cells at day 2 post-infection. WT mice were infected with HSV-2 ivag and vaginal cells were isolated at baseline through to day 3 post-infection. Vaginal cells were stained for CD45, CD3, NK1.1 CD27, and CD11b. Cells were first gated on CD45+, CD3–, and NK1.1+ to determine the NK cell population. NK cells were then examined for CD27 and CD11b expression. Representative flow plots are shown in **(A)**. Proportion of double-negative NK cells (CD27–CD11b–; Q4) is shown in (**B**; *n* = 4). Proportion of CD27+ (CD27+CD11b–; Q1) NK cells is shown in (**C**; *n* = 4). Proportion of double-positive (CD27+CD11b+; Q2) NK cells is shown in (**D**; *n* = 3). Proportion of CD11b+ (CD27–CD11b+; Q3) NK cells is shown in (**E**; *n* = 4). ^*^p < 0.05; ^**^p < 0.01; ^***^p < 0.001.

During a vaginal HSV-2 infection, we have previously shown that NK cells are activated to release an early wave of IFN-γ at day 2 post-infection (12, 21). Infection of WT mice with HSV-2 intravaginally yielded a rapid upregulation of IFN-γ at d2 post-infection as previously described. We also found a similarly rapid downregulation of IFN-γ at d3 post-infection (Supplementary Figure 2). Indeed, we observed an almost complete abrogation of IFN-γ levels at d3 post-infection. This would suggest that the activated NK cells are disappearing, becoming exhausted, or negatively regulated during infection. With this in mind, we examined the number of NK cells within the vaginal tract between the peak of IFN-γ production (day 2 post-infection) and the time point at which IFN-γ is abrogated (day 3 post-infection). We found very low levels of NK cells at baseline and 1 day after infection (Figures 1A,B). At day 2 post-infection, we observed a significant accumulation of NK cells in the vaginal mucosa that was maintained at day 3 post-infection (Figures 1A,B). This suggests that the abrogation in IFN-γ levels at d3 post-infection is not due to a reduction of NK cells within the vaginal tract. We then assessed whether a difference in NK cell maturation status could contribute to the difference in IFN-γ levels at d2 and d3 post-infection. We found that NK cells at baseline and d1 post-infection have a higher proportion of immature double negative NK cells (Q4) compared to NK cells at the peak of IFN-γ production (d2 p.i.; Figures 2A,B). There was no significant difference levels of CD27+CD11b– (Q1) or the double positive (Q2) NK cell populations at d2 and d3 post-infection (Figures 2A,C,D). We did find, however, a significant increase in the most mature NK cell population (CD27–CD11b+, Q3) at d2 post-infection in comparison to d3 post-infection (Figure 2E). Staining controls are shown in the supplementary data (Supplementary Figure 3). Despite finding similar numbers of NK cells at days 2 and 3 post-infection, there was a decrease in the proportion of mature CD27–CD11b+ (Q3) NK cells at d3 post-infection in comparison to d2 post-infection. Though there is a minor decrease in the most mature phenotype related to high NK cell effector function, this alteration in maturation status is unlikely to be solely responsible for the complete abrogation of NK cell IFN-γ production at d3 post-infection.

### Type I IFN Receptor on NK Cells Negatively Regulates Their Release of IFN-γ

**Figure 3:**
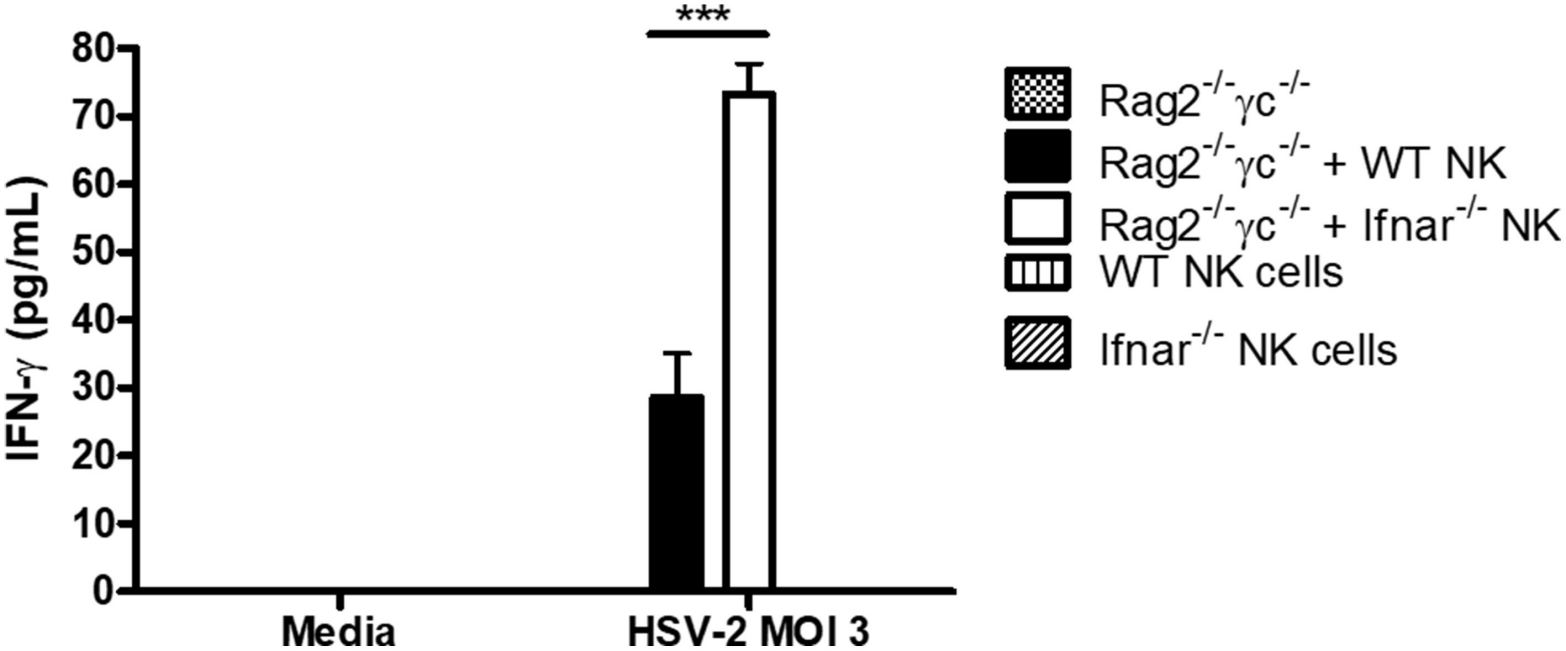
Absence of type I IFN receptor on NK cells allows for a significant increase in IFN-γ production from NK cells. Rag2^−/−^γc^−/−^ splenocytes were isolated and infected with HSV-2 MOI 3. Infected Rag2^−/−^γc^−/−^ splenocytes were then co-cultured with isolated WT or *Ifnar*^−/−^ NK cells for 48 h. As controls, Rag2^−/−^γc^−/−^ splenocytes, WT, and *Ifnar*^−/−^ NK cells were each infected in separate wells for 48 h. After 48 h of incubation, supernatants were collected and assayed for IFN-γ protein production (*n* = 3 repeated once with similar results). ^***^p < 0.001.

We had previously found that during HSV-2 infection, type I IFN receptor was required to activate NK cell IFN-γ production, but its action was not required directly on NK cells (12). Instead, type I IFN activated inflammatory monocytes to release IL-18, which was responsible for NK cell activation. We also found that transfer of NK cells lacking IFNAR into alymphoid *Rag2*^−/−^γ*c*^−/−^ mice yielded significantly increased levels of IFN-γ at both d2 and d3 post-infection in comparison to WT NK cells (12). This suggested to us that, in an *in vivo* setting, type I IFN receptor was required on NK cells in order to negatively regulate their IFN-γ response to virus infections. Here, we found that a similar phenomenon occurred in *vitro*, as HSV-2 infection of co-cultured WT or *Ifnar*^−/−^ NK cells with *Rag2*^−/−^γ*c*^−/−^ splenocytes led to significantly increased levels of IFN-γ from co-cultures containing *Ifnar*^−/−^ NK cells in comparison to WT NK cells (Figure 3). Overall, our *in vitro* data confirms that NK cells require type I IFN receptor in order to negatively regulate their release of IFN-γ during virus infection.

### Type I IFN Pre-treatment Dampens Cytokine-Induced NK Cell IFN-γ Production

**Figure 4:**
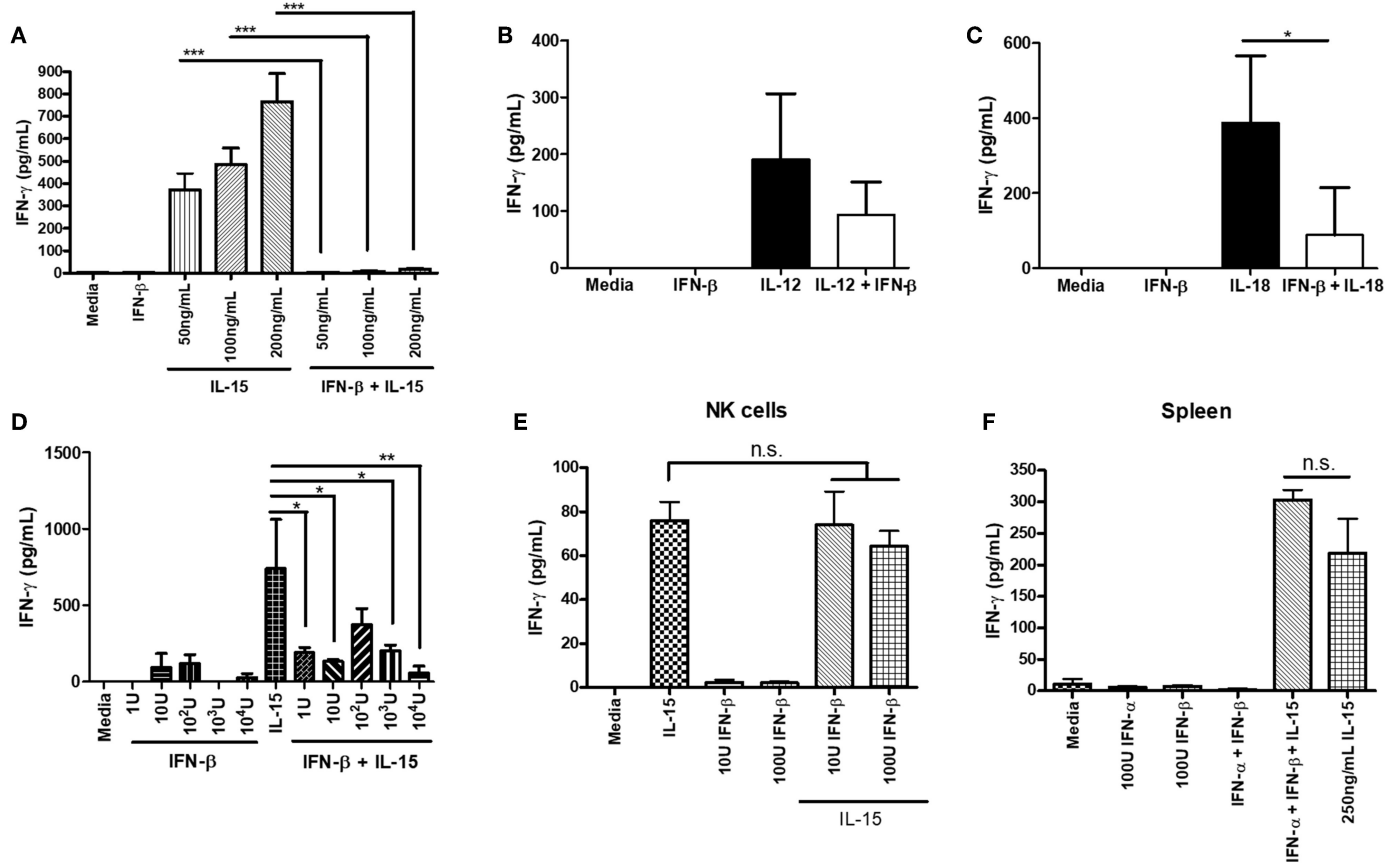
Type I IFN pre-treatment suppresses cytokine-induced IFN-γ production from NK cells. NK cells were isolated from WT spleens and pre-treated with 100 U of IFN-β for 12 h. After the 12 h pre-treatment, NK cells were stimulated with the indicated doses of IL-15 (**A**; *n* = 3, repeated once with similar results), 1 ng/mL IL-12 (**B**; *n* = 3), or 25 ng/mL IL-18 (**C**; *n* = 3, repeated once with similar results) for 24 h. Supernatants were collected and assayed for IFN-γ production. WT splenocytes were pre-treated with increasing doses of IFN-β for 4 h. After 4 h of pre-treatment, splenocytes were stimulated with 250 ng/mL of IL-15 for 24 h. Supernatants were collected and assayed for IFN-γ production (**D**; *n* = 3, repeated once with similar results). NK cells were isolated from WT spleen and stimulated with either 200 ng/mL IL-15 alone, different doses of IFN-β alone, or IL-15 and IFN-β at the same time for 24 h. Supernatants were assayed for IFN-γ (**E**; *n* = 3). WT splenocytes were isolated and stimulated with either 100 U IFN-α, 100 U IFN-β, 250 ng/mL IL-15, or a combination of IFN-α, IFN-β, and IL-15 at the same time. After 24 h of stimulation, supernatants were collected and assayed for IFN-γ levels (**F**; *n* = 3). n.s., not significant, ^*^p < 0.05, ^**^p < 0.01, ^***^p < 0.001.

We further explored the ability of type I IFN to suppress NK cell IFN-γ production outside of a virus infection model. We wanted to determine if the negative regulatory ability of type IFN was restricted to infection or could be observed with other stimuli. We pre-treated isolated NK cells with IFN-β for 12 h prior to stimulating with activating cytokines. Treatment of NK cells with IFN-β prior to cytokine stimulation completely abrogated their ability to produce IFN-γ in response to varying doses of IL-15 and reduced the level of IFN-γ after IL-12 and IL-18 treatment (Figures 4A–C). Further, we found that in the presence of other immune cells, pre-treating splenocytes with IFN-β was able to significantly reduce IL-15-induced IFN-γ production, even with low concentrations of IFN-β (1 U; Figure 4D). We were concerned that IFN-β treatment was reducing the survival of NK cells, as type I IFN can be a pro-apoptotic factor. We examined NK cell survival after 12 h of IFN-β treatment and did not observe a large difference in survival between media treatment (63% viable) and IFN-β treatment (59% viable; data not shown). We examined the impact of type I IFN on NK cell IFN-γ production when given at the same time as an activating cytokine stimulus. Treatment of both isolated NK cells and splenocytes with type I IFN and IL-15 at the same time yielded no difference in IFN-γ levels (Figures 4E,**F**, respectively). Overall, our data suggest that type I IFN pre-treatment, but not simultaneous treatment, is able to negatively regulate IFN-γ from NK cells in both a direct manner and in the presence of other immune cells. Further, the negative regulatory capacity of type I IFN spans across different stimulatory conditions.

### Type I IFN Pre-treatment Suppresses Human NK Cell IFN-γ Production

**Figure 5:**
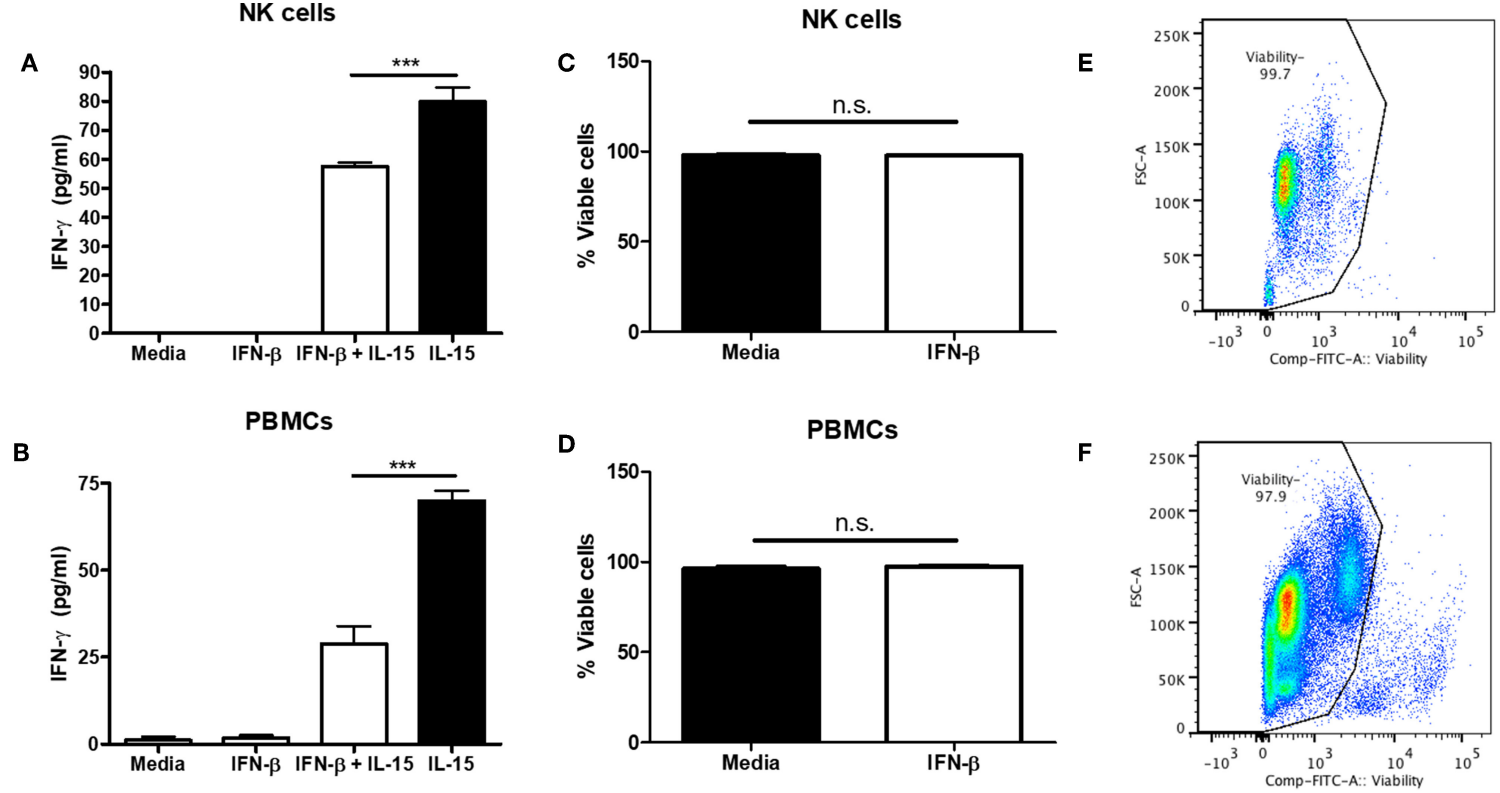
Type I IFN pre-treatment suppresses IL-15-induced human NK cell IFN-γ production. CD56+ cells were isolated from PBMCs and pre-treated with 100 U of IFN-β for 12 h. After 12 h, NK cells were then stimulated with 250 ng/mL IL-15 for 24 h. Supernatants were collected and assayed for human IFN-γ levels (**A**; *n* = 4). PBMCs were isolated and pre-treated with 100 U IFN-β for 12 h. After 12 h of treatment, PBMCs were stimulated with 250 ng/mL IL-15 for 24 h. Supernatants were collected and assayed for IFN-γ levels (**B**; *n* = 3). After 18 h of stimulation, cells were stained with a fixable viability dye. The proportion of viable cells is graphically shown (**C,D**, *n* = 3). Original flow plots are also shown for NK cells and PBMCs (**E,F**, respectively) ^***^p < 0.001.

Though we and others have found that Type I IFN is able to negatively regulate NK cell function, most of the data involves mouse modeling. Thus, we wanted to determine if type I IFN was able to negatively regulate human NK cells as well. PBMC-isolated NK cells were treated with IFN-β for 12 h prior to IL-15 stimulation. IFN-β pre-treatment significantly reduced NK cell IFN-γ production in comparison to IL-15 stimulation alone (Figure 5A). Moreover, pre-treatment of PBMCs with IFN-β significantly reduced IFN-γ in response to IL-15 stimulation (Figure 5B). To rule out the pro-apoptotic effects of type I IFN, we examined viability after 18 h of IFN-β treatment and observed no difference in viability between untreated and IFN-β-treated groups (Figures 5C–F). Our data suggest that type I IFN is able to negatively regulate human NK cell IFN-γ release. Further, we detected a greater IFN-β-induced reduction in IFN-γ levels in the presence of other immune cells.

### The Presence of Type I IFN Does Not Significantly Alter IL-10 Levels *in vitro* Nor *in vivo*

**Figure 6:**
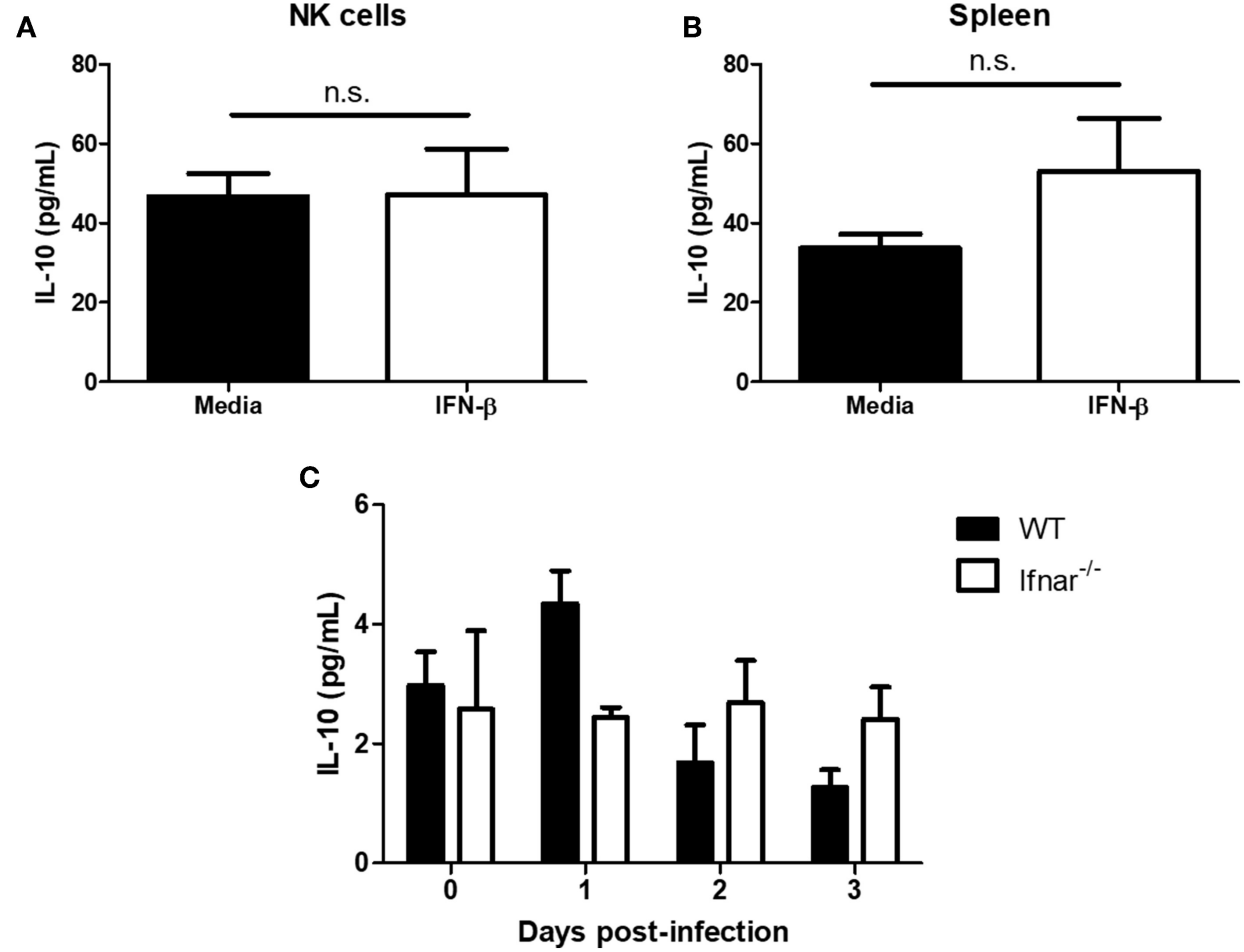
Type I IFN does not significantly alter levels of IL-10. Isolated WT splenocyte NK cells (**A**; *n* = 3, repeated once with similar results) and splenocytes (**B**; *n* = 3) were stimulated with media or IFN-β for 24 h. Supernatants were assayed for IL-10 production. WT and Ifnar^−/−^ mice were infected with HSV-2 ivag. Vaginal lavages were collected at baseline through to d3 p.i. and examined for IL-10 levels (**C**; *n* = 3). n.s., not significant.

Type I interferon has been shown to induce the immunosuppressive cytokine IL-10 during LCMV infection and mycobacterial infections (14, 17). During a mycobacterial infection, type I IFN has been shown to upregulate IL-10 and suppress IFN-γ production (17). Further, IL-10 has been shown to suppress NK cell IFN-γ production by acting on NK cells or suppressing maturation and/or activating cytokines (e.g., IL-12 and IL-18) from myeloid cells (22–25). From this, we wanted to determine whether type I IFN could mediate its immunosuppressive effects through the induction of IL-10. IFN-β treatment of isolated NK cells did not significantly alter IL-10 production in comparison to controls (Figure 6A). Further, there was no significant difference in IL-10 levels between splenocytes that were untreated or treated with IFN-β (Figure 6B). In the context of HSV-2 infection, we did not detect a significant alteration in IL-10 levels from WT vaginal lavages between different days post-infection. Similarly, we did not detect a significant difference in levels of IL-10 between WT and *Ifnar*^−/−^ mice (Figure 6C). This indicates that IL-10 is unlikely mediating the immunosuppression of IFN-γ production from NK cells in both the *in vitro* environment and during an *in vivo* HSV-2 infection.

### Type I IFN Stimulation Upregulates Axl Expression on NK Cells

**Figure 7:**
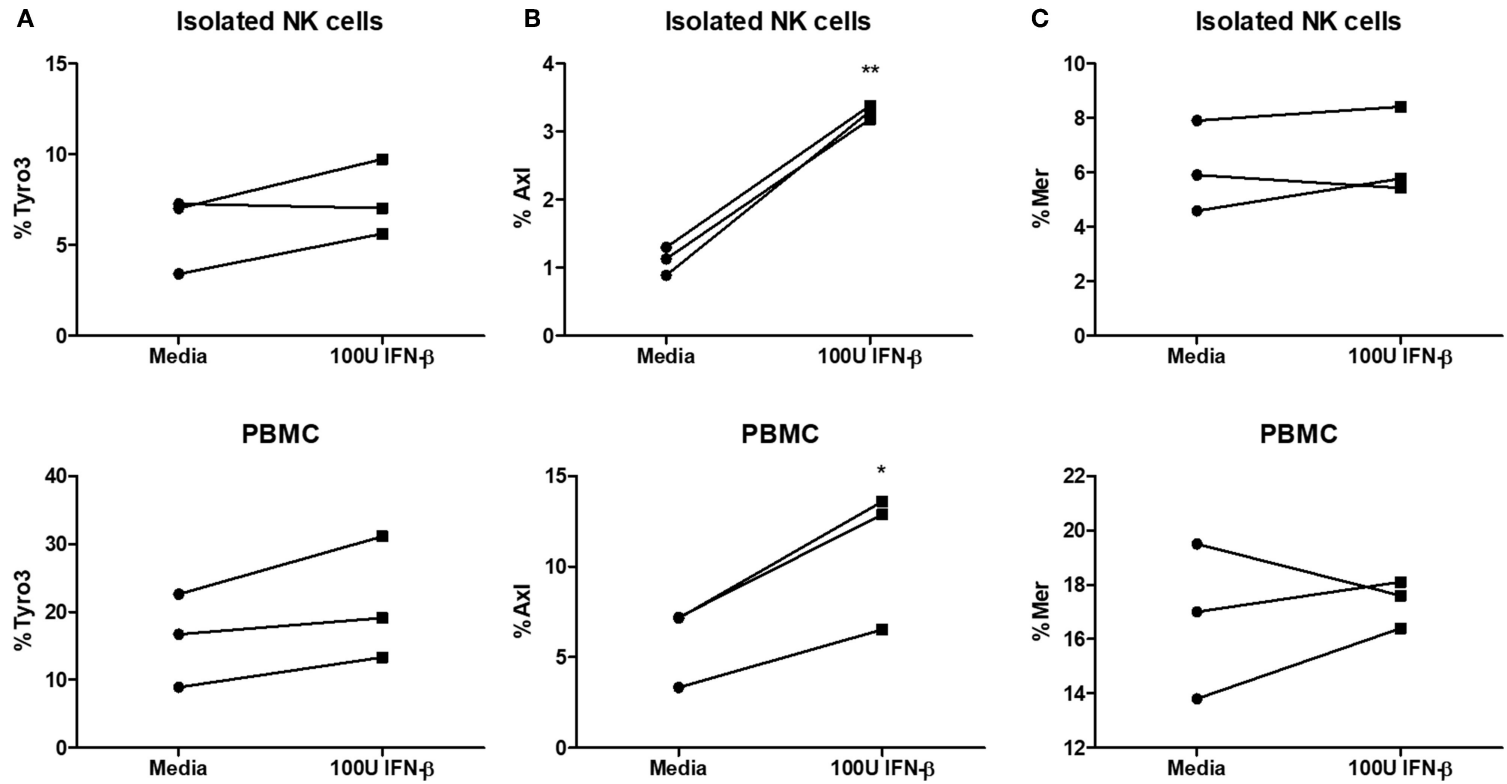
Significantly increased expression of Axl on NK cells after type I IFN stimulation. PBMC-isolated NK cells and PBMCs were stimulated with 100 U of IFN-β for 18 h. After stimulation, cells were stained with anti- CD56, CD3, Tyro3, Axl, and Mer. Cells were first gated as CD56+CD3– NK cells and then examined for expression of Tyro3 (**A**; *n* = 3), Axl (**B**; *n* = 3), or Mer (**C**; *n* = 3), ^*^p < 0.05, ^**^p < 0.01.

Type I IFN has been shown to upregulate TAM receptors (Tyro3, Axl, and Mer) on immune cells (18). The expression of TAM can redirect type I IFN signaling to induce the expression of suppressor of cytokine signaling (SOCS) proteins, which are known to suppress pro-inflammatory signaling pathways downstream of TLR and cytokine activation (18). We examined Tyro3, Axl, and Mer expression on NK cells after stimulating NK cells alone or PBMCs with IFN-β. We found no significant difference in Tyro3 expression on NK cells following type I IFN stimulation of either NK cells alone or PBMCs (Figure 7A). We did detect a significant upregulation of Axl expression on NK cells after stimulation of NK cells or PBMCs with IFN-β (Figure 7B). Similar to Tyro3, we found no difference in NK cell Mer expression after IFN-β stimulation in either the NK cell or PBMC group (Figure 7C). Overall, our data suggest that type I IFN stimulation can upregulate Axl expression on human NK cells.

## Discussion

We had previously found that type I IFN was critical for the activation of IFN-γ release from NK cells during HSV-2 infection (21). We determined that type I IFN activates inflammatory monocytes to release IL-18, which was necessary for activating NK cells (12). We also found that expression of type I IFN receptor on NK cells was required for the negative regulation of IFN-γ production during infection (12). We demonstrated that mouse *Ifnar*^−/−^ NK cells had significantly increased and sustained levels of IFN-γ production during HSV-2 infection. In WT mice, we normally observe a peak of NK cell IFN-γ levels at d2 post-infection and an abrogation of IFN-γ at day 3 post-infection. Our data suggests that this down-regulation of IFN-γ is mediated by type I IFN rather than a loss of mouse NK cells at d3 post-infection as there was no difference in vaginal mouse NK cell number between d2 and d3 post-infection. Though we did find a significantly increased proportion of mature (CD27–CD11b+) mouse NK cells at d2 post-infection in comparison to d3 post-infection, this partial alteration in maturation is unlikely to be solely responsible for the abrogation of IFN-γ production at d3 post-infection. Pre-treatment of both mouse and human isolated NK cells and splenocytes (or PBMCs) with type I IFN suppressed cytokine-induced IFN-γ production. In trying to resolve the mechanism by which type I IFN negatively regulates NK cells, we found that there was no difference in IL-10 production between untreated mouse cells and cells treated with type I IFN. Similarly, we found no difference in IL-10 production between WT and *Ifnar*^−/−^ mice during HSV-2 infection. We did, however, detect a significant increase in human NK cell Axl expression after type I IFN treatment of both human NK cells and PBMCs.

In our studies, we showed that type I IFN can suppress IFN-γ release from both NK cells in isolation and in the presence of other immune cells. There have been several mechanisms by which type I IFN can suppress NK cell function reported in the literature, including the induction of external mediators or through direct modulation of NK cells themselves. In terms of direct modulation of NK cells by type I IFN, we detected a significant increase in Axl expression on NK cells after stimulating both isolated human NK cells and PBMCs with type I IFN. Evidence has shown that type I IFN is able to upregulate TAM receptor expression on immune cells, which can suppress their function as TAM receptors hijack the type I IFN signaling pathway to induce activation of SOCS proteins to repress antiviral function (18). Another mechanism through which TAM receptors regulate NK cell IFN-γ is through the recruitment of the E3 ubiquitin ligase Cbl-b, which inhibits NK cell activation signaling. *Cblb*^−/−^ NK cells demonstrated a heightened IFN-γ response upon stimulation through NKG2D (26). Other mechanisms of NK cell modulation include the interplay between STAT4 and STAT1 (27). It is well-known that STAT4 is required for induction of IFN-γ from NK cells. Miyagi et al. found that STAT4 was preferentially associated with the type I IFN receptor and was displaced by STAT1 upon type I IFN stimulation to dampen IFN-γ production (27). Further, Nguyen et al. demonstrated that type I IFN inhibition of NK cell function was dependent upon STAT1 (15).

While in the presence of other immune cells, IL-10 and PD ligand 1 (PDL1) have been thought to play a role in the effects of type I IFN immunoregulation. During a chronic virus infection, such as LCMV, type I IFN suppresses IFN-γ production with blockade of the type I IFN receptor restoring the antiviral IFN-γ response (14). This was shown to be mediated by IL-10 (14). Moreover, type I IFN has also been shown to induce IL-10 production during *Mycobacterium leprae* infections (17). In contrast to this, however, we found no difference in IL-10 production when cells were stimulated with type I IFN. Additionally, there was no significant alteration in IL-10 expression between WT and *Ifnar*^−/−^ mice infected with HSV-2. Type I IFN has also been found to upregulate PDL1 on CD8α+ and CD8α- DCs (14). Hsu et al. has shown that PDL1 can also suppress NK cell function by ligating with the PD receptors on NK cells (28). In another avenue, Cousens et al. found that both IFN-α and IFN-β suppressed IL-12 production, which is a potent stimulator of NK cell IFN-γ production (29). Further, others have found that type I IFN can suppress IL-12 production from DCs and IL-12p40 production from PBMCs (30, 31). While IL-10 may not play a role in the suppression of IFN-γ from NK cells, there remain several mechanisms by which this suppression might occur both through modulation of NK cells themselves or through external mediators. There may be a multitude of pathways by which type I IFN is able to immunosuppress NK cell function. As we did observe an upregulation of Axl expression in human NK cells in response to type I IFN stimulation, understanding whether Axl mediates the negative regulation of IFN-γ through downstream SOCS or Cbl-b signaling will be an important area of future study.

Recent evidence suggests that the subtypes of type I IFN can have very different modulatory effects on immune cells. While all subtypes can potently induce an antiviral response, Garcin et al. found that treating DCs with different IFN subtypes led to significantly different profiles of receptor expression and cytokine production (32). Further, the timing of type I IFN production as well as the array of immune cells present during type I IFN production can impact the effect of type I IFN on a specific immune cell (16). During HSV-2 infection, an initial wave of IFN-β occurs between 6 and 12 h post-infection, followed by a wave of IFN-α production at 48 h post-infection (21, 33). The wave of IFN-α production at 48 h post-infection could be responsible for negatively regulating NK cell IFN-γ production during HSV-2 infection as this coincides with the peak of NK cell recruitment to the vaginal tract.

In summary, our data suggest that type I IFN can directly suppress NK cell IFN-γ production during HSV-2 infection and cytokine stimulation. We found that type I IFN does not alter IL-10 production, however, it does increase expression of Axl receptor on NK cells, which has previously been shown to suppress immune cell function. As this finding is correlative at this point, it will be informative to assess the interplay between type I IFN, Axl and NK cell function. Importantly, we demonstrate that type I IFN does not directly activate NK cells, rather, it negatively regulates their IFN-γ production. Collectively, our data provide evidence for a new fundamental understanding of how type I IFN directly and indirectly modulates NK cell function.

It is important to understand how NK cell function is negatively regulated in order to prevent dysregulated function and limit immunopathology. In the context of HSV-2 infection, Singh et al. (34) showed that while asymptomatic individuals produced higher levels of IFN-γ from their PBMCs compared to individuals with recurrent genital ulcers, addition of IFN-γ to HSV-2 infected cells from recurrent individuals led to heightened virus replication, rather than a reduction (34). In addition, systemic administration of IFN-γ prior to endotoxin administration has been shown to worsen mortality from toxic shock, while IFN-γ overexpression has been shown to contribute to the development of systemic autoimmune diseases, including SLE (35, 36).

Overall, our findings contribute to a larger understanding for the role of type I IFN in modulating the NK cell response to virus infection. A theme of balance is emerging, where IFN-γ requires tight control to both optimize the antiviral impact of this cytokine, but to also limit its detrimental consequences. Too much can lead to immunopathology, while too little favors virus replication. From our studies, it appears that type I IFN plays a central role in this process.

## Ethics Statement

This study was carried out in accordance with the recommendations of the Canadian Council on Animal Care guidelines. The protocol was approved by McMaster University's Animal Research Ethics Board. This study was carried out in accordance with the recommendations of the Hamilton Integrated Research Ethics Board at McMaster University. All subjects gave written informed consent in accordance with the Declaration of Helsinki. The Protocol was approved by the Hamilton Integrated Research Ethics Board.

## Author Contributions

AL organized and carried out the majority of the experiments, completed the data analysis, and wrote the manuscript. FM, SP, MS, TC, and MC all contributed to the experimental design and completion. AA is the corresponding author, guided experimental design, and edited the manuscript.

### Conflict of Interest Statement

The authors declare that the research was conducted in the absence of any commercial or financial relationships that could be construed as a potential conflict of interest.

## Supplementary Material

Supplementary Figure 1 Purity of NK1.1+ cells from the NK1.1+ magnetic selection kit. NK cells were isolated from splenocytes using the NK1.1 magnetic selection kit from Stem Cell Technologies and examined for NK1.1 expression.

Supplementary Figure 2 HSV-2 induced IFN-γ production is upregulated at day 2 post-infection and abrogated at day 3 post-infection. WT mice were infected with HSV-2 ivag. Days 0–3 p.i. vaginal lavages were collected and assayed for IFN-γ (*n* = 5). ^***^*p* < 0.001.

Supplementary Figure 3 Staining controls for CD27 and CD11b. WT mice were infected with HSV-2 ivag. Vaginal cells were isolated at baseline through to day 3 post-infection. A splenocyte control was also isolated at the same time. Vaginal cells and splenocytes were stained with anti-CD45, CD3, NK1.1 CD27, and CD11b. Cells were first gated on CD45+, CD3, and NK1.1 to determine the NK cell population. NK cells were then examined for CD27 and CD11b expression. CD27 and CD11b FMO staining controls are shown. Splenocytes stained and gated with the same strategy are also shown.
