# Supplementary figures and images for: Type I Interferon Receptor on NK Cells Negatively Regulates Interferon-γ Production
Source: Front Immunol. 2019 Jun 4;10:1261. doi: 10.3389/fimmu.2019.01261 (PMC6558015; doi:10.3389/fimmu.2019.01261)

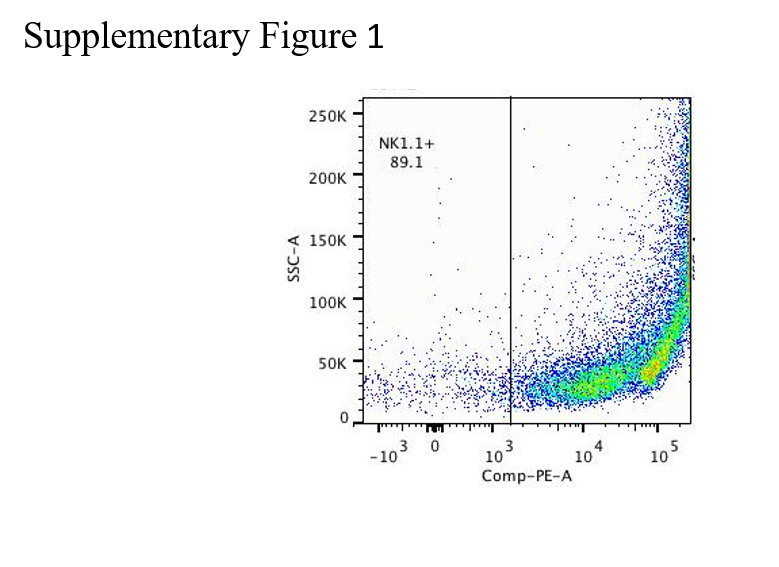

Supplement: Supplementary Figure 1 — Purity of NK1.1+ cells from the NK1.1+ magnetic selection kit. NK cells were isolated from splenocytes using the NK1.1 magnetic selection kit from Stem Cell Technologies and examined for NK1.1 expression. [file Image_1.TIF]

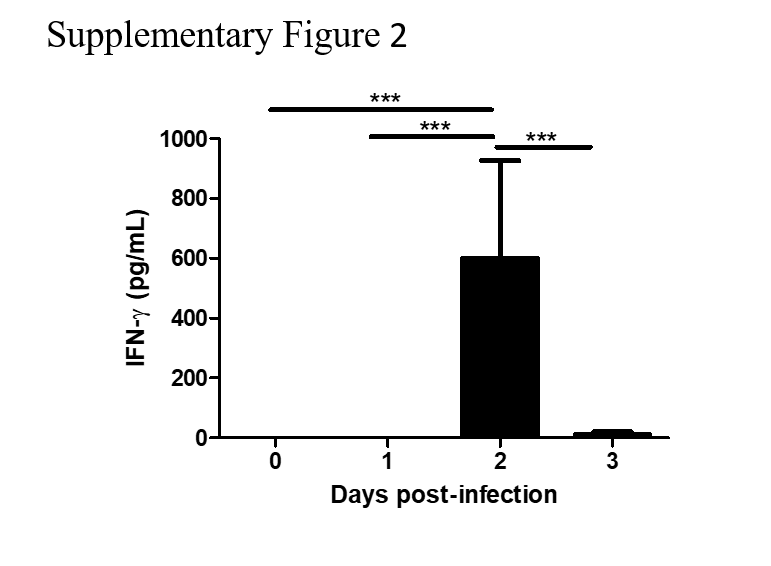

Supplement: Supplementary Figure 2 — HSV-2 induced IFN-γ production is upregulated at day 2 post-infection and abrogated at day 3 post-infection. WT mice were infected with HSV-2 ivag. Days 0–3 p.i. vaginal lavages were collected and assayed for IFN-γ (n = 5). ***p < 0.001. [file Image_2.TIF]

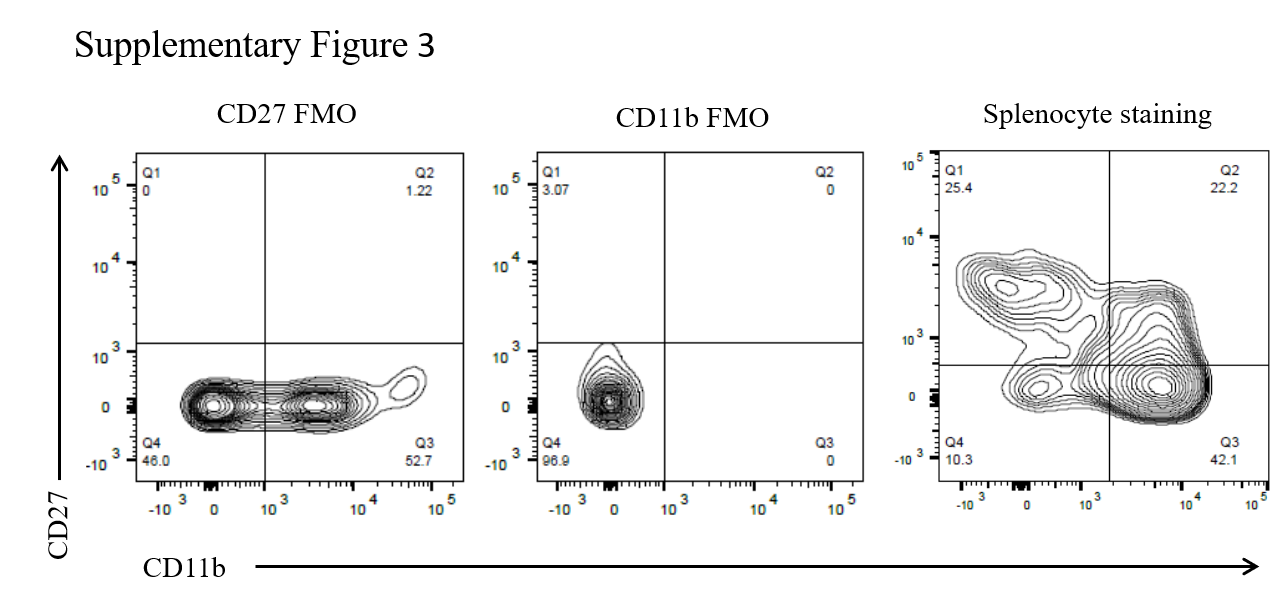

Supplement: Supplementary Figure 3 — Staining controls for CD27 and CD11b. WT mice were infected with HSV-2 ivag. Vaginal cells were isolated at baseline through to day 3 post-infection. A splenocyte control was also isolated at the same time. Vaginal cells and splenocytes were stained with anti-CD45, CD3, NK1.1 CD27, and CD11b. Cells were first gated on CD45+, CD3, and NK1.1 to determine the NK cell population. NK cells were then examined for CD27 and CD11b expression. CD27 and CD11b FMO staining controls are shown. Splenocytes stained and gated with the same strategy are also shown. [file Image_3.TIF]
